# IcsA autotransporter passenger promotes increased fusion protein expression on the cell surface

**DOI:** 10.1186/1475-2859-11-20

**Published:** 2012-02-07

**Authors:** Mabel Lum, Renato Morona

**Affiliations:** 1 Australian Bacterial Pathogenesis Program, Discipline of Microbiology and Immunology, School of Molecular and Biomedical Science, University of Adelaide, Adelaide, South Australia, Australia

## Abstract

**Background:**

Autotransporters are attractive cell surface display vehicles as they lack complex adaptor proteins necessary for protein export. Recent reports have suggested that the native effector domain (α domain) and translocation domain (β domain) interact with each other to drive translocation of the effector domain to the outer membrane. In this report we compared the expression, surface localisation and folding of TEM-1 β-lactamase (Bla) and maltose binding protein (MalE or MBP) fused to either full length *Shigella flexneri *IcsA (IcsA) autotransporter or to the β domain alone (IcsA_β_) to determine the contribution of the native IcsA α domain in presenting the fusion proteins on the surface of *E. coli *K-12 UT5600 (Δ*ompT*).

**Results:**

Expression of IcsA-Bla was greater than IcsA_β_-Bla. High levels of IcsA-MalE were detected but IcsA_β_-MalE was not expressed. All fusion proteins other than IcsA_β_-MalE were localised to the outer membrane and were detected on the surface of UT5600 via immunofluorescence microscopy. All bacteria expressing IcsA-MalE were labelled with both α-IcsA and α-MBP. UT5600 expressing IcsA_β_-MalE was not labelled with α-MBP. A third of UT5600 expressing IcsA-Bla were detectable with α-Bla but only 5% of UT5600 (IcsA_β_-Bla) were labelled with α-Bla. The correct folding of the Bla moiety when fused to IcsA and IcsA_β _was also retained as UT5600 expressing either fusion protein exhibited a decreased zone of inhibition in the presence of ampicillin. UT5600 expressing IcsA-Bla was more resistant compared to UT5600 expressing IcsA_β_-Bla.

**Conclusions:**

The export mechanism of autotransporters is not well understood but accumulating evidence suggest a critical role for the native effector or α domain in facilitating its own export via interactions with the translocation or β domain. This is the first report directly comparing expression of heterologous proteins fused to the full length IcsA autotransporter and fusion to the β domain alone. Protein expression and surface presentation of the fusion proteins were dramatically improved when fused to IcsA rather than IcsA_β_. Future studies involved in designing autotransporters as cell surface display vehicles would benefit from including the native α domain. This work also provides further evidence for a key interaction between the autotransporter α and β domains.

## Background

Expression of heterologous proteins and peptides on bacterial surface are important for a wide range of applications such as vaccine production, bioremediation, biocatalysis, peptide library screening and translocation studies [1]. The autotransporter or type Va secretary pathway is an attractive cell surface display vehicle due to the lack of complex adaptor machineries necessary for protein export compared to other Gram negative export pathways [2]. High expression of recombinant molecules (> 10^5^) per cell without adverse effects on cell viability have been reported [3]. Fusion proteins displayed on bacterial surface can be subjected to FACS (fluorescence-activated cell sorting) and ELISA (enzyme-linked immunosorbent assay) to facilitate high throughput screenings [4]. The strict genotype to phenotype linkage of the autotransporter system aids phenotypic selections [5]. Furthermore autotransporters can also be expressed in other Gram negative bacteria making it highly versatile [6,7]. Epitopes displayed have been reported to elicit strong immune stimulation, making it a suitable medium for potential live vaccine development [8].

Cell surface display with autotransporters has its drawbacks. Disulphide bond formation within the exogenous protein has been reported to affect protein translocation but this has been addressed by inactivating *dsbA*, a dithiol oxidase, which assists in disulphide bond formations in periplasmic proteins [7,9,10]. The successful translocation of the single chain immunoglobulin fragment with a disulfide bridge to the bacterial surface [11] suggest that the final dimension of the heterologous protein is the limiting factor rather than the presence of the disulfide bridge. OmpT, an outer membrane (OM) protease, has been shown to cleave exogenous proteins after translocation, thus it is important to use a host strain with an *ompT *deletion [7,9].

The autotransporter protein IcsA (or VirG) is a key virulence factor of *Shigella flexneri*. It is polarly distributed on the OM and is essential for mediating *Shigella*'s intra- and intercellular motility in the host colonic epithelium through activation of the host neural Wiskott-Aldrich syndrome protein (N-WASP) and subsequent actin nucleation through the Arp2/3 complex [12-16]. The 120 kDa IcsA protein is a typical autotransporter with three distinct regions; an N-terminal signal sequence (amino acids (aa) 1-52), a central effector domain or α domain (aa 53-758) and a C-terminal translocation domain or β domain (aa 759-1102) [17]. The translocation of the IcsA polypeptide from the cytoplasm to the periplasm is directed by its unusually long signal sequence via the Sec pathway [18]. The signal sequence is also crucial in maintaining the stability of fusion constructs [19]. In the periplasm, an intramolecular disulphide bridge is formed in the IcsA α domain [20]. The 80 kDa α domain is exported to the extracellular milieu when the 37 kDa β domain inserts itself into the OM [17]. The translocation unit of NalP (*Neisseria meningitides*), EspP (*E. coli *strain O157:H7) and the trimetric EstA (*Pseudomonas aeruginosa*) autotransporters are made up of a 12 stranded C-terminal β-barrel pore with a N-terminal α-helix inserted into the pore itself [21-23]. The IcsA translocation unit is likely to adopt a similar confirmation with a predicted N-terminal α-helix [15]. IcsA_α _can be cleaved on the surface by its specific protease IcsP (SopA) between Arg_758_-Arg_759 _[24-26].

In most examples of autotransporter-based display systems, the native α domain is replaced with the peptide or protein of interest and is expressed as a single polypeptide with the β domain [3,6,15]. Previously the FimH lectin domain was fused to a portion of the *E. coli *Antigen 43 (Ag43) autotransporter α domain and was exported to the cell surface by the Ag43 β domain. The chimeric protein was expressed, its function retained and was presented on the surface of the *Salmonella enterica *host strain [6]. This suggest that the presence of the α domain, at least a portion of it is not detrimental to the stability of the chimeric polypeptide.

Recently Saurí et al. suggested that the native α and β domains of ATs form specific interactions which facilitate efficient translocation of the α domain [27]. Replacement of the haemoglobin protease (Hbp) β domain with the closely related EspP β domain, resulted in the secretion of the Hbp α domain, albeit with reduced efficiency [27]. Increasing or decreasing the size of the Hbp β domain by modifying the number of β strands also affected translocation of the α domain [27]. Furthermore the conserved residues of the α-helix embedded inside the EspP β domain is not crucial for efficient translocation but is indispensable for proteolytic release of the Hbp α domain [28].

In this report, the stability and expression in *E. coli *K-12 UT5600 (Δ*ompT*) of TEM-1 β-lactamase (Bla) and *E. coli *maltose binding protein (MalE or MBP) fused to either the full length IcsA protein (IcsA) or to the β domain alone (IcsA_β_) was compared to determine the contribution of the α domain to the stability, expression and surface localisation of the chimeric proteins. The outcomes of this study will aid in future cell surface display design and also in improving our understanding of the autotransporter export pathway.

## Results & discussion

### Construction of IcsA and IcsA_β _fusion proteins

**Figure 1:**
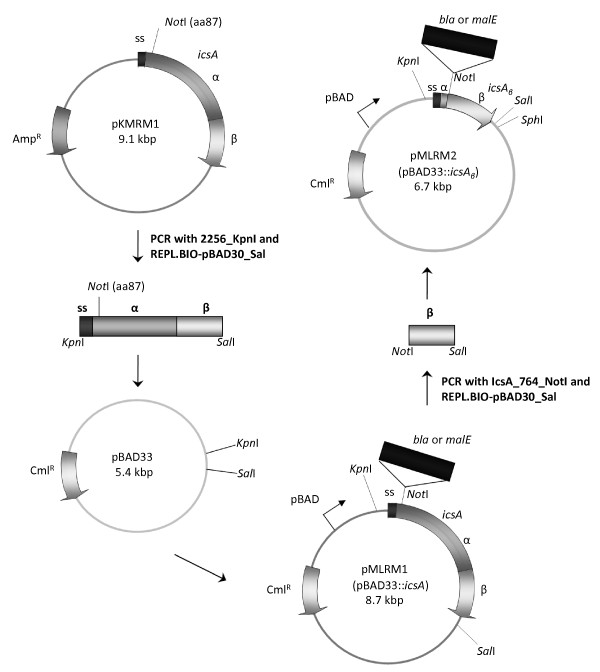
**Schematic representation of the construction of pMLRM1 and pMLRM2**. *icsA *was amplified from pKMRM1 and was cloned into pBAD33, resulting in pMLRM1. *icsA_β _*was amplified from pMLRM1 and was cloned into pBAD33, resulting in pMLRM2. The *Not*I site (TGCGGCCGC: amino acid 88-91, nucleotide 262-270) of *icsA *and *icsA_β _*serves as the insertion site for *bla *(TEM-1 beta lactamase) and *malE *(*E. coli *maltose binding protein). pBAD is the arabinose inducible promoter, ss represents the IcsA signal peptide, α represents the IcsA passenger domain and β represents the IcsA translocator domain. Drawing is not to scale.

In a previous study, a *Not*I site was inserted into *icsA*, as part of a 15 nucleotide in-frame insertion at amino acid 87 (*icsA_i87_*, TGCGGCCGCAATGGA: amino acid 88-92, nucleotide 261-270) [29]. *icsA_i87 _*will herein be referred as *icsA. icsA *was cloned via digestion with *EcoR*I and *Sal*I with likewise digested pBAD33 to generate pMLRM1 (Figure 1). In a previous study, IcsA_β _(amino acid 764-1102) was fused to maltose binding protein (MalE/MBP) and alkaline phosphatase to dissect the IcsA export pathway [15]. The C-terminal β-barrel pore and the embedded N-terminal α-helix, herein referred as *icsA_β _*(amino acid 764-1102), was amplified from pMLRM1 with a forward primer incorporating a *Not*I restriction site and a reverse primer incorporating a *Sal*I restriction site. *icsA_β _*was cloned via digestion with *Not*I and *Sal*I with likewise digested pBAD33 to generate pMLRM2. The expression of the *icsA *and *icsA_β _*coding regions in pMLRM1 and pMLRM2, respectively, is under the control of the tightly regulated arabinose-inducible *araBAD *promoter [30].

**Figure 2:**
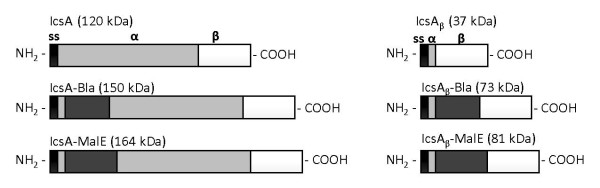
**Summary of IcsA and IcsA_β _fusion proteins**. Black boxes represent the IcsA signal peptide (ss), light grey boxes represent the 80 kDa IcsA_α _(α) and white boxes represent the 37 kDa IcsA_β _(β). TEM-1 beta lactamase (Bla) and maltose binding protein (MalE/MBP) are represented by dark grey boxes. Predicted molecular weights are shown in parentheses.

IcsA and IcsA_β _proteins fused to the 29 kDa Bla and 44 kDa MalE were generated to investigate the contribution of the IcsA passenger domain to the overall stability of the chimeric proteins (Figure 2). Both Bla and MalE are relatively globular and have been used in various autotransporter studies [15,31]. The *bla *and *malE *coding regions were subcloned from pKMRM1 and pMAL-c2, respectively, with forward and reverse primers incorporating a *Not*I restriction site resulting in pMLRM28 (pMLRM1::*bla*), pMLRM20 (pMLRM2::*bla*), pMLRM39 (pMLRM1::*malE*) and pMLRM37 (pMLRM2::*malE*). Figure 1 outlines the construction of these plasmids. The plasmids were transformed into an *ΔompT E. coli *K-12 strain, UT5600, to prevent fusion protein cleavage by OmpT, an OM protease [7,9].

### Effects of pMLRM1 and pMLRM2 fusion protein expression on the growth of *E. coli *K-12 UT5600

The effects of inducing the pBAD promoter in pMLRM1::*bla *and pMLRM2::*bla *were compared to pMLRM1 and pBAD33 in the *E. coli *K-12 UT5600 strain. The growth rates of UT5600 carrying pMLRM1, pMLRM1::*bla *and pMLRM2::*bla *were comparable to UT5600 carrying pBAD33 (data not shown, Additional file 1: Figure S1).

### Expression of chimeric IcsA and IcsA_β _fusion proteins

Production of the chimeric IcsA-Bla, IcsA_β_-Bla, IcsA-MalE and IcsA_β_-MalE in UT5600 were examined by SDS-PAGE and Western immunoblotting after arabinose induction. At every 30 min, a 1 mL sample was taken and kept on ice before they were standardised to 1 × 10^8 ^bacteria/mL with 2× sample buffer. All pMLRM1 chimeric constructs were immunoblotted with both α-IcsA and also the respective specific antibodies (α-Bla, α-MBP). All pMLRM2 chimeras were only blotted with the respective specific antibodies since our anti-IcsA serum does not recognise the β domain of IcsA.

**Figure 3:**
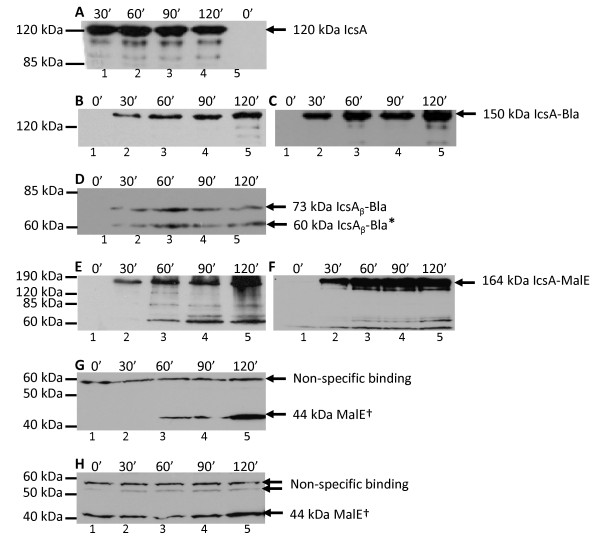
**Analysis of IcsA/IcsA_β _fusion protein production**. UT5600 strains were grown to mid-log phase and induced with 0.2% (w/v) arabinose. Samples were taken at times indicated. Approximately 1 × 10^8 ^bacteria were solubilised, electrophoresed on a 12% SDS-polyacrylamide gel and subjected to Western immunoblotting. Immunoblotting of (**A**) MLRM4 (pMLRM1) with anti-IcsA; (**B**) MLRM28 (pMLRM1::*bla*) with anti-IcsA and (**C**) anti-Bla; (**D**) MLRM20 (pMLRM2::*bla*) with anti-Bla; (**E**) MLRM39 (pMLRM1::*malE*) with anti-IcsA and (**F**) anti-MBP; (**G**) MLRM37 (pMLRM2::*malE*) with anti-MBP; (**H**) UT5600 with anti-MBP. The apparent molecular sizes of the proteins are indicated. *Protein degradation products ^†^MalE induced due to chromosomal gene in UT5600

IcsA induced from UT5600 (pMLRM4) was immunoblotted with α-IcsA as shown in Figure 3A. The amount of IcsA detected did not appear to be increase over the 2 h time course; the smaller MW bands were probably IcsA breakdown products.

IcsA-Bla induced from UT5600 (pMLRM28) was immunoblotted with α-IcsA and α-Bla (Figure 3B and 3C). Increasing amounts of proteins were detected over the time course of pMLRM28 induction. Small amounts of degraded proteins were observed after 2 h (Figure 3B and 3C, lane 5). When IcsA_β_-Bla induced from UT5600 (pMLRM20) was imunoblotted with α-Bla (Figure 3B), low levels of IcsA_β_-Bla was detected without any increase in protein levels over time. A smaller 60 kDa IcsA_β_-Bla was also consistently observed.

IcsA-MalE induced from UT5600 (pMLRM39) was detected by immunoblotting with α-IcsA and α-MBP (Figure 3E and 3F). Increasing amounts of the fusion protein were detected over the time course of pMLRM39 induction. Degraded IcsA-MalE proteins appeared after 1 h induction of pMLRM39 arabinose induction, consistent with the observed increased amount of protein production (Figure 3E and 3F, lanes 3-5). When IcsA_β_-MalE induced from UT5600 (pMLRM37) was immunoblotted with α-MBP (Figure 3G), the 81 kDa IcsA_β_-MalE was not detected. Instead two bands of approximately 44 kDa and 60 kDa were observed.

It was expected that the expression of all constructs subcloned into the pBAD33-based vectors would increase when the strains were induced with arabinose. This was observed for IcsA-Bla (Figure 3B and 3C). In the case of IcsA_β_-Bla, the expression level of the protein remained constant over a period of 2 h (Figure 3D). The consistency of the protein levels induced from the pBAD promoter was also observed when IcsA was induced from pMLRM1 (Figure 3A).

Due to the rapid induction of the pBAD promoter, it is likely that the maximal protein induction has been reached by 30 min. Consistent with this hypothesis, degraded proteins were observed for both 120 kDa IcsA (Figure 3A, lanes 1-4) and 150 kDa IcsA-Bla (Figure 3B and 3C, lane 5). The sizes of the degraded proteins were greater than 85 kDa in both cases. Compared to IcsA-Bla, very low levels of the 73 kDa IcsA_β_-Bla was expressed (Figure 3D). The presence of a smaller 60 kDa protein suggests degradation of IcsA_β_-Bla was rapid and smaller polypeptides were subsequently not detected by Western immunoblotting. It can be concluded that Bla fused to IcsA resulted in higher levels of but not necessarily more stable chimeras compared to fusion to IcsA_β_.

Over the 2 h induction with arabinose, increasing amounts of proteins, both full length and degraded (≥ 60 kDa) were observed for IcsA-MalE (Figure 3E and 3F, lanes 3-5). IcsA-MalE was cleaved either during or after translocation from the periplasm to the OM. No IcsA_β_-MalE was observed by whole cell western immunoblotting even though a slower induction at 25°C was attempted (not shown). Increasing amounts of a 44 kDa protein, corresponding to MalE was present at all time points after arabinose induction (Figure 3G). As MalE expression was induced in UT5600 alone by the presence of arabinose (Figure 3H), hence the 44 kDa MalE protein was background MalE expression by UT5600. The lack of IcsA_β_-MalE expression cannot be explained at this stage. No mutations were identified in DNA sequencing, and previous attempts to detect MalE fusions to the translocation domain of IcsA and other autotransporters have been successful [15,32].

### OM localisation

To determine if the fusion chimeras were presented on the surface of UT5600, two different approaches were undertaken; cell fractionation and immunofluorescence (IF) microscopy.

The UT5600 strains (MLRM28 - pMLRM1::*bla*, MLRM20 - pMLRM2::*bla*, MLRM39 - pMLRM1::*malE*, MLRM37 - pMLRM2:*:malE*) were subjected to cell fractionation to determine if the IcsA and IcsA_β _chimeras were located in the OM.

**Figure 4:**
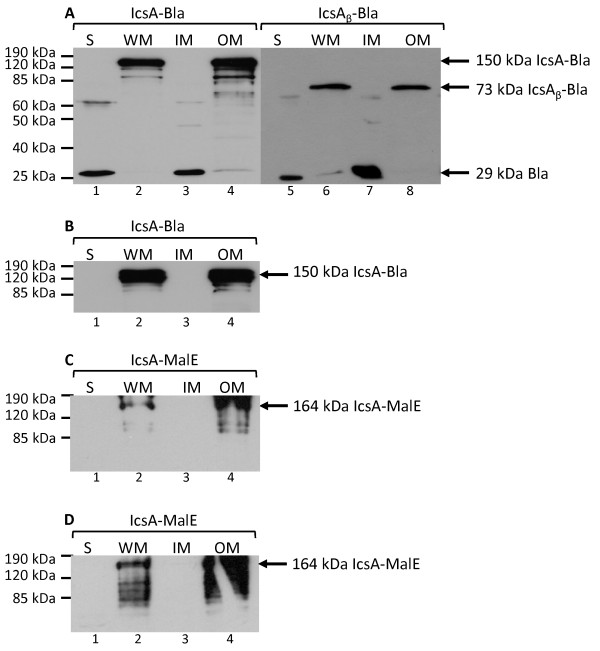
**Detection of IcsA and IcsA_β _fusion proteins in the OM**. UT5600 strains were grown to mid-log phase, induced with 0.2% (w/v) arabinose and were fractionated into soluble fraction (S), whole membrane (WM), inner membrane (IM) and outer membrane (OM). Equivalent amounts of protein extracts from each fraction were loaded and analysed by Western immunoblotting. Immunobloting of fractions from **(A)** MLRM28 (pMLRM1::*bla*) and MLRM20 (pMLRM2::*bla*) with anti-Bla; **(B)** MLRM28 (pMLRM1::*bla*) with anti-IcsA; **(C) **MLRM39 (pMLRM1::*malE*) with anti-MBP and **(D)** anti-IcsA. The apparent molecular sizes of the proteins are indicated.

The 150 kDa band corresponding to IcsA-Bla was detected in the whole membrane (WM) and OM when immunoblotted with α-Bla and α-IcsA (Figure 4A and 4B, lanes 2 and 4). IcsA_β_-Bla was also detected in the WM and OM (Figure 4A, lanes 6 and 8). A band corresponding to the size of the mature 29 kDa Bla was observed in the soluble fraction (S) and the inner membrane (IM) of UT5600 strains expressing IcsA-Bla (MLRM28) and IcsA_β_-Bla (MLRM20) (Figure 4A, lanes 1, 3, 5 and 7). The association of the Bla protein at the IM has not been reported previously and is most likely due to an artefact from fractionation. Degraded proteins (< 120 kDa) were observed for IcsA-Bla (Figure 4A and 4B, lanes 2 and 4) but not IcsA_β_-Bla in the WM and OM fractions (Figure 4A, lanes 6 and 8).

IcsA-MalE (pMLRM1::*malE*) was detected in both the WM and OM fractions of UT5600 when immunoblotted with α-IcsA and α-MBP (Figure 4C and 4D, lanes 2 and 4). MalE-related bands (> 70 kDa) were also observed, similar to immunoblotting results following induction with arabinose (Figure 3E and 3F).

### Surface localisation

**Figure 5:**
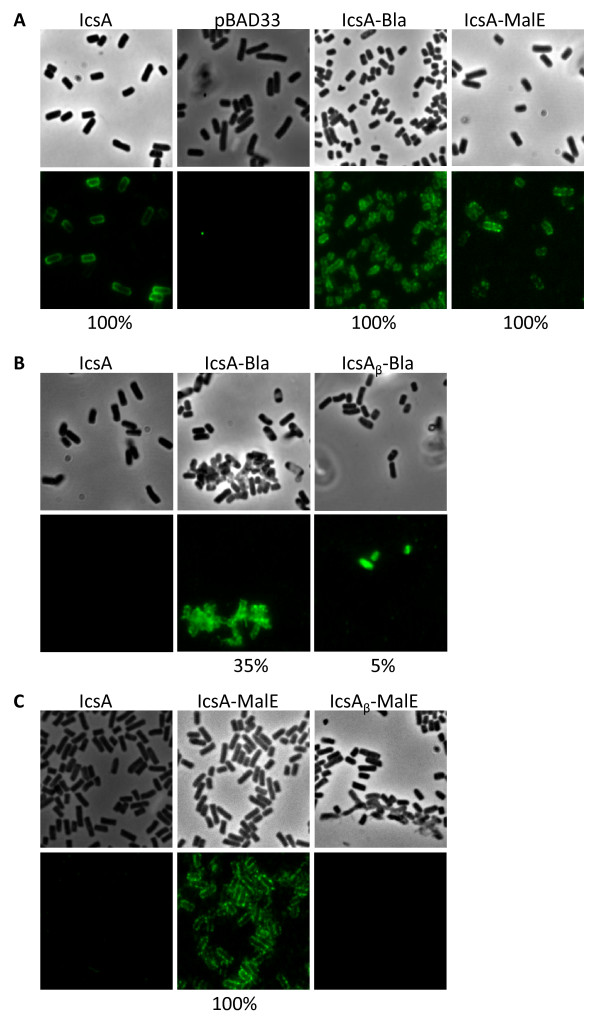
**Detection of IcsA and IcsA_β _fusion proteins on the surface of UT5600 strains**. Immunofluorescence microscopy showing surface presentation of pBAD33 (empty vector) (MLRM3), IcsA (MLRM4), IcsA-Bla (MLRM28), IcsA_β_-Bla (MLRM20), IcsA-MalE (MLRM39) and IcsA_β_-MalE (MLRM37) with (**A**) anti-IcsA; (**B**) anti-Bla; (**C**) anti-MBP. Phase contrast microscopy (upper panels) and fluorescence microscopy (lower panels) were performed. Specific proteins were detected with their respective antibodies and were detected by FITC-labelled anti-rabbit immunoglobulin serum. The relative percentage of FITC-positive cells are indicated (n = 100)

IF microscopy was carried out to determine if the chimera IcsA and IcsA_β _were presented on the surface of UT5600. The chimeric IcsA autotransporters were detected with α-IcsA (Figure 5A). As expected the positive control (pMLRM4 - pBAD33::*icsA*) was detected on the cell surface on both the lateral and polar regions of UT5600. The negative control for α-IcsA was UT5600 (pBAD33) (pMLRM3) and cell surface labelling was not observed. UT5600 strains expressing IcsA-Bla and IcsA-MalE were labelled with α-IcsA on both the lateral and polar regions, consistent with the OM localisation observations from cell fractionation (Figure 4B and 4D). UT5600 strains expressing Bla chimeras were immunostained with α-Bla to determine if the Bla moiety was presented on the surface of UT5600 (Figure 5B). UT5600 strain expressing IcsA (pMLRM4) was not labelled with α-Bla, as expected. Both Bla chimeras, IcsA-Bla and IcsA_β_-Bla were detected on the lateral and polar regions of UT5600. The staining of UT5600 (pMLRM28 - IcsA-Bla) (35% of bacteria labelled) was greater than UT5600 (pMLRM20 - IcsA_β_-Bla) (5% of bacteria labelled). However compared to α-IcsA staining (Figure 5A), fewer IcsA-Bla expressing cells were detected with α-Bla, suggesting that the Bla moiety was cleaved, either during or after transport from the cytoplasm. The low level of IcsA_β_-Bla detected for UT5600 (pMLRM20 - IcsA_β_-Bla) was not surprising, given that a low level IcsA_β_-Bla was detected by Western immunoblotting (Figure 3D).

UT5600 strains expressing MalE chimeras (pMLRM39 - IcsA-MalE and pMLRM37- IcsA_β_-MalE) were immunostained with α-MBP to determine if MalE was presented on the bacterial surface (Figure 5C). As expected the negative control, UT5600 (pMLRM4 - pBAD33::*icsA*) was not labelled with α-MBP. IcsA-MalE was detected on both the lateral and polar regions of UT5600 (pMLRM39) in all the cells, similar to labelling with α-IcsA (Figure 5A). None of UT5600 (pMLRM37) expressing IcsA_β_-MalE had reacted with α-MBP.

### Activity of bla fusion proteins

**Figure 6:**
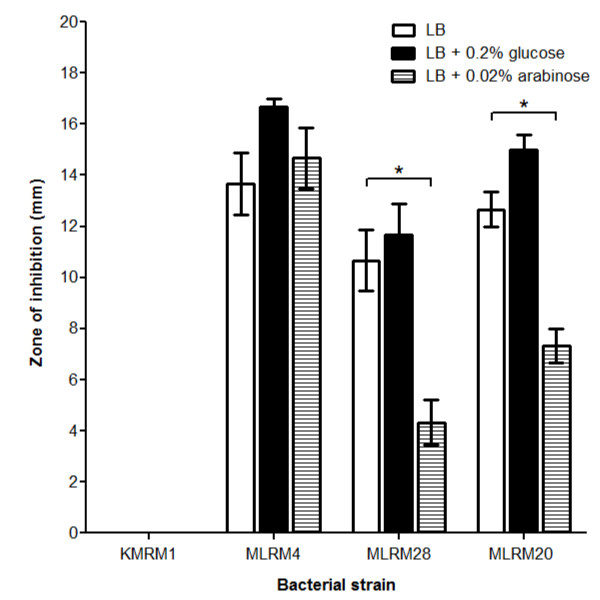
**Ampicillin susceptibility of UT5600 strains expressing IcsA and IcsA_β _fusion proteins are resistant to 150 μg ampicillin**. Bacterial strains expressing Bla (KMRM1), IcsA (MLRM4), IcsA-Bla (MLRM28) and IcsA_β_-Bla (MLRM20) were added to 5 mL soft agar and were overlaid onto three different solidified media: LB alone, LB + 0.2% glucose or LB + 0.2% arabinose. 150 μg ampicillin was spotted onto sterile Whatman filters, and the agar plate was incubated at 37°C for 16 h. The diameter of the zone of inhibition was measured in millimetre (mm). KMRM1 is resistant to ampicillin. Data are represented as mean ± SEM of three independent experiments. *p < 0.05

The activity of the Bla fusion proteins was assessed by measuring the ampicillin resistance of MLRM28 (IcsA-Bla) and MLRM20 (IcsA_β_-Bla). Bacterial cells were mixed with soft agar and were overlaid on solidified media. The diameter of the zone of bacterial inhibition in the presence of 150 μg ampicillin was measured. Mean values representing three independent experiments and SEM are presented in Figure 6.

The ampicillin resistance of MLRM28 (IcsA-Bla) and MLRM20 (IcsA_β_-Bla) were compared to MLRM4 (IcsA) and KMRM1, the original strain from which *bla *was originally amplified from in three different conditions: LB alone, LB with 0.2% (w/v) glucose and LB with 0.02% (w/v) arabinose. Glucose represses the pBAD promoter, hence the resistance of the Bla constructs when grown on LB agar should be similar to LB with 0.2% (w/v) glucose agar. Higher resistance levels towards ampicillin was anticipated for bacterial cultures overlaid on 0.02% (w/v) arabinose plates.

Strain KMRM1 expressing Bla displayed 100% resistance towards ampicillin and was not affected by the growth media. No observable differences in resistance were noted for Bla fusion proteins grown on LB and LB + 0.2% (w/v) glucose, as expected. MLRM28 (IcsA-Bla) was more resistant to ampicillin compared to MLRM20 (IcsA_β_-Bla). This is not surprising given that more IcsA-Bla was detected via Western immunoblotting compared to IcsA_β_-Bla (Figure 3B, C and 3D). Furthermore a third of UT5600 expressing IcsA-Bla was presented on the bacterial surface compared to 5% of cells expressing IcsA_β_-Bla (Figure 5B). Overall the ability of the UT5600 strains expressing IcsA-Bla and IcsA_β_-Bla to grow in the presence of ampicillin suggest proper folding of the Bla moiety and retention of enzymatic activity when fused to IcsA and IcsA_β_.

## Conclusions

The export pathway of autotransporters is not well understood but recent evidence suggests that the native effector domain plays a critical role in facilitating its own translocation when it traverses through the translocation domain via interactions with specific residues [27]. A direct comparison between heterologous proteins fused to full length autotransporter and heterologous proteins fused to the translocation domain has previously not been reported. In this study, protein expression and surface localization of Bla and MalE fused to full length IcsA and IcsA_β _alone was compared.

The overall data suggest that fusion of exogenous proteins close to the amino terminal of the IcsA autotransporter passenger domain significantly improves protein expression and surface presentation when compared to chimeras fused directly to the IcsA translocation domain. Differences in protein expression, OM localisation and surface presentation between chimera proteins of full length IcsA and IcsA_β _was to some extent dramatic. For both Bla and MalE, expression of IcsA fusions was greater than IcsA_β _chimeras. However the overall expression of IcsA_β _itself could not be assessed, therefore its contribution to the lack of IcsA_β _chimera expression cannot be ruled out. Surface presentation of IcsA chimeras were also significantly greater compared to IcsA_β _as determined by IF microscopy. Degraded MalE and Bla fusion proteins suggest that some degree of protein degradation occurred during protein export. Nonetheless the Bla protein was highly active as UT5600 (MLRM28 - pMLRM1::*bla*) and UT5600 (MLRM20 - pMLRM2::*bla*) showed resistance towards ampicillin.

Overall, the results here suggest that the autotransporter display system can be improved dramatically by fusing proteins to the native passenger domain, providing further evidence for specific interactions between the α and β domain in protein translocation.

## Methods

### Bacterial strains, plasmids and growth conditions

The strains and plasmids used in this study are listed in Table 1. *Escherichia coli *K-12 strain DH5α was used for routine cloning and *E. coli *K-12 strain UT5600 was used as host cells for the surface display of fusion proteins. All bacterial strains were routinely cultured in Luria Bertani (LB). Bacteria were grown in media with antibiotics for 16 h with aeration and then subcultured 1/20 and grown to mid-exponential growth phase by incubation with aeration for 2 h at 37°C. Bacterial strains harbouring pBAD33 and derivatives were cultivated in LB broth with 0.2% (w/v) glucose. Where appropriate, media were supplemented with ampicillin (100 μg/mL) and chloramphenicol (25 μg/mL).

**Table 1:** Bacterial strains and plasmids

Strain or plasmid'	Relevant characteristics	Reference or source
*E. coli *K-12		
DH5α	F^- ^φ80*lac*ZΔM15 Δ(*lac*ZYA-*arg*F)U169 *rec*A1 *end*A1 *hsd*R17(rk^-^, mk^+^) *pho*A*sup*E44 *thi*-1 *gyr*A96 *rel*A1 λ-	Gibco-BRL
KMRM1	DH5α [pKMRM1]; Amp^R^	[29]
MLRM3	UT5600 [pBAD33]; Cm^R^	This study
MLRM4	UT5600 [pMLRM1]; Cm^R^	This study
MLRM20	UT5600 [pMLRM2::*bla *(*Not*I - *Not*I)], Cm^R^	This study
MLRM28	UT5600 [pMLRM1::*bla *(*Not*I - *Not*I)], Cm^R^	This study
MLRM37	UT5600 [pMLRM2::*malE *(*Not*I - *Not*I)], Cm^R^	This study
MLRM39	UT5600 [pMLRM1::*malE *(*Not*I - *Not*I)], Cm^R^	This study
RMA3045	E1315 [pMAL-c2], Amp^R^	Laboratory collection
UT5600	F^- ^*ara-14 leuB6 secA6 lacY1 proC14 tsx-67 Δ(ompT-fepC)266 entA403 trpE38 rfbD1 rpsL109 xyl-5 mtl-1 thi-1*	Laboratory collection
Plasmids		
pBAD33	pBAD expression; pACYC184 ori; Cm^R^	[30]
pIcsA	*icsA (EcoR*I - *Sal*I) gene cloned into pBR322; medium copy no.; ColE1 *ori*; Amp^R^	[33]
pKMRM1	pIcsA::5aa insertion at amino acid position 87 (*icsA_i87 _*or *icsA*) (*bla*); Amp^R^	[29]
pMLRM1	*icsA *(*Kpn*I - *Sal*I) gene cloned into pBAD33 (pBAD33::*icsA*), Cm^R^	This study
pMLRM2	*icsA_ß _*(amino acid 764-1102) (*Not*I - *Sal*I) cloned into pMLRM1 (pBAD33::*icsA_β_*); Cm^R^	This study
pMAL-c2	pMAL-c2 (*malE*); Amp^R^	New England Biolabs

### Plasmid construction and DNA methods

In a previous study, a *Not*I site was inserted into *icsA*, as part of a 15 nucleotide in-frame insertion at aa 87 [*icsA*, TGCGGCCGCAATGGA: amino acid 88-92, nucleotide 261-270] [29]. The *icsA *gene was amplified by PCR from pKMRM1 with primers 2156_KpnI (5'-GCCGGTACCAACGGAATCTTTTCAGGGG) and REPL.BIO-pBAD30_Sal (5'-CACGCCCTGTCGACTTATTATCAGAAGG) containing *Kpn*I and *Sal*I sites, respectively. The resulting PCR product was digested with *Kpn*I and *Sal*I, and ligated into the *Kpn*I and *Sal*I sites of the pBAD33 plasmid to produce pMLRM1 (Figure 1).

The *icsA_β _*region of *icsA *was amplified by PCR from pMLRM1 with primers IcsA_764_NotI (5'-CTAGCGGCCGCCTTGTATCTTCAC) and REPL.BIO-pBAD30_Sal containing *Not*I and *Sal*I sites, respectively. The PCR product was digested with *Not*I and *Sal*I and ligated between the *Not*I and *Sal*I sites of the pMLRM1 plasmid to produce pMLRM2. The *bla *gene was amplified by PCR from a plasmid preparation of pKMRM1 with primers Bla_NotI_f (5'-CTGGCGGCCGCCACCCAGAAACGC) and Bla_NotI_rev3 (5'-GTCGCGGCCGCCCCAATGCTTAATCAGTGAG) containing *Not*I sites. The resulting PCR product was digested with *Not*I and ligated into the *Not*I site of the pMLRM1 and pMLRM2 plasmids to produce pMLRM28 and pMLRM20, respectively. The *malE *gene was amplified by PCR from a plasmid preparation of pMAL-c2 with primers MalE_NotI_f (5'- GACGCGGCCGCAAAATCGAAGAAGGTA) and MalE_NotI_rev5 (5'-CGAGCGGCCGCCAGTCTGCGCGTCTTTC) containing *Not*I sites. The resulting PCR product was digested with *Not*I and ligated into the *Not*I site of the pMLRM1 and pMLRM2 plasmids to produce pMLRM39 and pMLRM37, respectively. DNA sequencing was used to confirm the integrity of the inserts within the constructed plasmids. Isolation of plasmid DNA was carried out with the QIAprep Miniprep kit (Qiagen). Restriction endonucleases were used according to the manufacturer's specifications (NEB). General cloning techniques, PCR and DNA sequencing were performed as described previously [29].

### Antibodies and antisera

Affinity-purified rabbit polyclonal anti-IcsA antiserum was as described previously [34]. Rabbit α-Bla polyclonal antibody was from Chemicon, and rabbit α-MBP antiserum was from NEB. Anti-IcsA was used at 1:1000, and anti-Bla and anti-MBP were used at 1:10,000.

### Measuring growth rate

Ten milliliters 18 h bacterial culture were subcultured (1/20) into LB + 0.2% (w/v) glucose and were grown for 1 h, washed in 2 × 20 mL pre-warmed fresh LB before induction with 0.2% (w/v) arabinose in a side arm flask in an orbital (200 rpm) water bath at 37°C. A spectrophotometer (Spectronic 20 D+, Milton Roy) was used to take absorbance readings at 600 nm every 30 min until the ABS_600 _reading reached 1.999. The experiment was repeated twice.

### SDS-PAGE and western immunoblotting

Ten milliliters bacterial cultures were subcultured (1/20) for 2 h, 37°C in LB + 0.2% (w/v) glucose, washed once in 20 mL fresh LB before induction with 0.2% (w/v) arabinose for 2 h at 37°C. 1 mL culture was centrifuged (13,000 rpm, 1 min, Eppendorf 5415R) and was standardised to 1 × 10^8 ^bacteria/mL with 2× sample buffer (0.125 M Tris-HCl, pH 6.8, 4% [w/v] SDS, 20% [v/v] glycerol, 10% [v/v] β-mercaptoethanol, 0.04% [w/v] Bromophenol blue). SDS-PAGE and Western immunoblotting were carried out as described previously [29]. Molecular weight markers used were BenchMark™ Pre-Stained Protein Ladder (Invitrogen).

### Cell fractionation

One hundred milliliters bacterial cultures were subcultured (1/20) for 2 h, 37°C in LB + 0.2% (w/v) glucose and were pelleted by centrifugation (8,000 rpm, 15 min, 4°C, Beckman Model J2-21 M). Cultures were washed in 2 × 20 mL fresh LB and induced with 0.2% (w/v) arabinose for 2 h at 37°C. Bacteria were pelleted by centrifugation (8,000 rpm, 15 min, 4°C, Beckman Model J2-21M), pellet washed with 3 mL 50 mM Tris-HCl (pH 8.0) and resuspended in 10 mL HEPES/1 mM MgCl_2_. Cultures were passaged through a French press and cell debris was pelleted by centrifugation (4,500 rpm, 15 min, RT, Sigma 3K15). The supernatant was pelleted by centrifugation (35,000 rpm, 1 h, 4°C, Beckman Coulter Optima™ L-100 Ultracentrifuge). A sample of the pellet (WM - whole membrane) was kept and resuspended in 100 μL 2× sample buffer. 10 μL of the supernatant (S/N) (soluble fraction) was mixed with 100 μL 1× sample buffer. The WM pellet was resuspended in 2.5 mL Triton/MgCl_2 _and was incubated at RT, shaking for 30 min. The mixture was pelleted by centrifugation (35,000 rpm, 1 h, 4°C, Beckman Coulter Optima™ L-100 Ultracentrifuge). A sample of the pellet (outer membrane) was kept and resuspended in 100 μL 2× sample buffer. 10 μL of the S/N (inner membrane) was mixed with 100 μL 1× sample buffer. Proteins were subjected to SDS-PAGE and detected via Western immunoblotting with specific antibodies.

### Indirect immunofluorescence (IF) of whole bacteria

Ten milliliters bacterial cultures were subcultured (1/20) for 2 h, 37°C in LB + 0.2% (w/v) glucose and were pelleted by centrifugation (13,000 rpm, 1 min, Eppendorf, 5415 R). Cultures were resuspended in 20 mL fresh LB and were pelleted by centrifugation (13,000 rpm, 1 min). Bacterial cultures were resuspended in 10 mL LB + 0.2% (w/v) arabinose and were induced for 2 h at 37°C. 500 μL of bacteria were pelleted by centrifugation (13,200 rpm, 1 min) and the supernatant discarded. Bacteria were fixed in 500 μL formalin (3.7% paraformaldehyde in 0.85% saline) for 30 min at room temperature. Fixed bacteria were pelleted by centrifugation (13,000 rpm, 1 min) and were resuspended in 500 μL phosphate-buffered saline (PBS). Fixed bacteria were pelleted by centrifugation (13,000 rpm, 1 min) and were resuspended in 100 μL PBS. Sterile cover-slips were placed in a 24-well tray and were incubated with filter sterilised poly-L-lysine (0.01% (w/v) in PBS) for 1 min and was aspirated. The cover-slips were washed with PBS before 4 μL of formalin fixed bacteria were centrifuged onto the cover-slips (2,500 rpm, 5 min, Heraeus Labofuge 400 R). The bacteria were washed twice in PBS and were incubated with the specific primary antibody diluted 1:100 with 10% foetal calf serum (FCS). Bacteria were washed thrice in PBS and incubated with Alexa 488-conjugated donkey anti-rabbit secondary antibody (Molecular Probes) in PBS with 10% FCS. Cover slips were washed thrice in PBS were mounted on glass slides with Mowiol 4-88 (Calbiochem) containing 1 mg/mL *p*-phenylenediamine (Sigma) and examined with an Olympus IX-70 microscope with phase-contrast optics using a 100× oil immersion objective and a 1.5× enlarger as required. Fluorescence and phase contrast images were false colour merged using the Metamorph software program (version 6.3r7; Molecular Devices).

### Ampicillin susceptibility testing

Ten milliliters bacterial cultures were subcultured (1/20) for 2 h, 37°C in LB + 0.2% (w/v) glucose and were pelleted by centrifugation (13,000 rpm, 1 min, Eppendorf, 5415 R). Cultures were resuspended in 20 mL fresh LB and were pelleted by centrifugation (13,000 rpm, 1 min). Bacterial cultures were resuspended in 10 mL LB + 0.2% (w/v) arabinose and were induced for 2 h at 37°C. 100 μL of bacteria (5 × 10^7 ^cells total) were added to the 5 mL soft agar (1:1 LB agar to LB broth, 56°C), swirled before it was overlayed onto pre-warmed LB agar (42°C) and allowed to set. 6 mm sterile Whatman filters were placed onto the agar and were soaked with a 150 μg ampicillin solution. The filter was allowed to dry before incubation at 37°C for 16 h. The diameter of the zone of inhibition was measured. The experiment was repeated twice.

### Statistical analysis

Statistical analysis was carried out using GraphPad Prism 5. Results are expressed as means ± SEM of data obtained in independent experiments. Statistical differences between groups were determined with two-tailed unpaired *t*-test. Statistical significance was set at *p *< 0.05.

## Competing interests

The authors declare that they have no competing interests.

## Authors' contributions

ML performed all the experimental work and wrote to the manuscript. RM conceived of the study, and participated in its design and coordination and helped to draft the manuscript. All authors read and approved the final manuscript.

## Supplementary Material

Additional file 1 **Figure S1**. Expression of IcsA-Bla and IcsA_β_-Bla does not affect UT5600 growth. The growth of UT5600 strains carrying pMLRM1::*bla *(MLRM28) and pMLRM2::*bla *(MLRM20) were comparable to pBAD33 (MLRM3) and pMLRM1 (MLRM4). An absorbance reading at 600 nm for each culture was taken every 30 min until the reading reached 1.999. Data are represented as mean ± SEM of three independent experiments. Click here for file
