# Supplementary figures and images for: IcsA autotransporter passenger promotes increased fusion protein expression on the cell surface
Source: Microb Cell Fact. 2012 Feb 7;11:20. doi: 10.1186/1475-2859-11-20 (PMC3298707; doi:10.1186/1475-2859-11-20)

## Slide 1
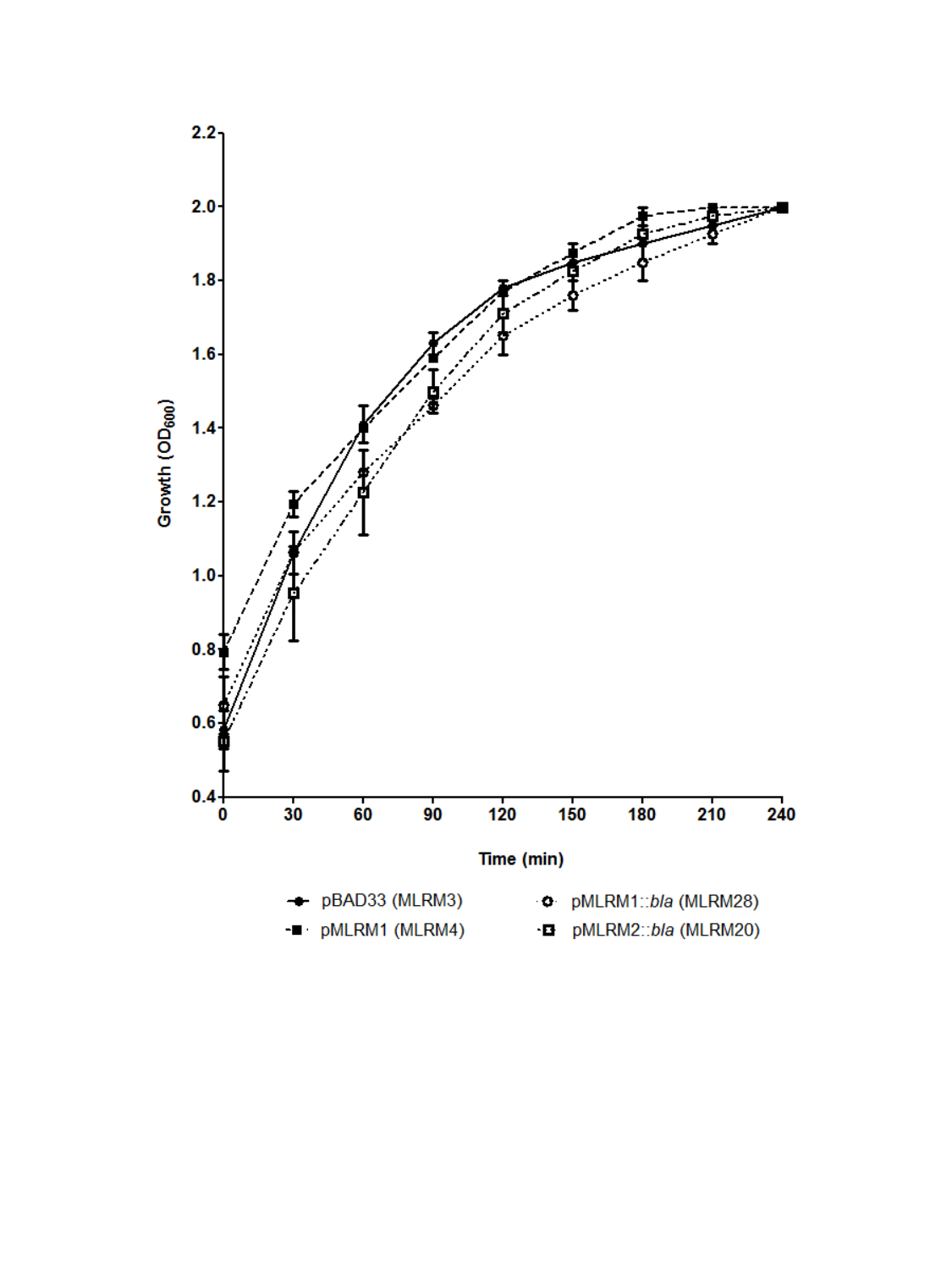

Supplement: Additional file 1 — Figure S1. Expression of IcsA-Bla and IcsAβ-Bla does not affect UT5600 growth. The growth of UT5600 strains carrying pMLRM1::bla (MLRM28) and pMLRM2::bla (MLRM20) were comparable to pBAD33 (MLRM3) and pMLRM1 (MLRM4). An absorbance reading at 600 nm for each culture was taken every 30 min until the reading reached 1.999. Data are represented as mean ± SEM of three independent experiments. [file 1475-2859-11-20-S1.PPT]
